# *In situ* self-assembly of amphiphilic dextran micelles and superparamagnetic iron oxide nanoparticle-loading as magnetic resonance imaging contrast agents

**DOI:** 10.1093/rb/rbac096

**Published:** 2022-12-05

**Authors:** Linrui Jiang, Rong Zheng, Ni Zeng, Changqiang Wu, Hongying Su

**Affiliations:** Faculty of Chemical Engineering, Kunming University of Science and Technology, Kunming 650500, China; Faculty of Chemical Engineering, Kunming University of Science and Technology, Kunming 650500, China; Faculty of Chemical Engineering, Kunming University of Science and Technology, Kunming 650500, China; Sichuan Key Laboratory of Medical Imaging, North Sichuan Medical College, Nanchong 637000, China; Faculty of Chemical Engineering, Kunming University of Science and Technology, Kunming 650500, China

**Keywords:** micelle, dextran, superparamagnetic iron oxide, magnetic resonance imaging, Schiff base

## Abstract

Polymeric micelles have long been considered as promising nanocarrier for hydrophobic drugs and imaging probes, due to their nanoscale particle size, biocompatibility and ability to loading reasonable amount of cargoes. Herein, a facile method for dextran micelles preparation was developed and their performance as carriers of superparamagnetic iron oxide (SPIO) nanocrystals was evaluated. Amphiphilic dextran (Dex-*g*-OA) was synthesized via the Schiff base reactions between oxidized dextran and oleylamine, and self-assembled *in situ* into nano-size micelles in the reaction systems. The self-assembling behaviors of the amphiphilic dextran were identified using fluorescence resonance energy transfer technique by detection the energy transfer signal between the fluorophore pairs, Cy5 and Cy5.5. Hydrophobic SPIO nanoparticles (Fe_3_O_4_ NPs) were successfully loaded into the dextran micelles via the *in situ* self-assembly process, leading to a series of Fe_3_O_4_ NPs-loaded micelle nanocomposites (Fe_3_O_4_@Dex-*g*-OA) with good biocompatibility, superparamagnetism and strongly enhanced *T*_2_ relaxivity. At the magnetic field of 0.5 T, the Fe_3_O_4_@Dex-*g*-OA nanocomposite with particle size of 116.2 ± 53.7 nm presented a higher *T*_2_ relaxivity of 327.9 ${\text{mM}}_{\text{Fe}}^{-1}$·s^−1^. The prepared magnetic nanocomposites hold the promise to be used as contrast agents in magnetic resonance imaging.

## Graphical abstract

**Figure:**
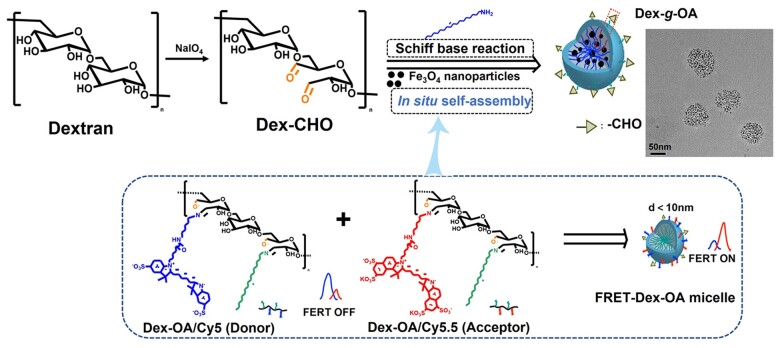

## Introduction

Polymeric micelles, which were formed by self-assembling of amphiphilic polymers in aqueous solution, have drawn significant attention as promising nanocarriers for poor water-soluble drug and imaging probe delivering [1–3]. Take advantages of their unique nanosized core/shell structure, hydrophobic drugs or nanoparticles can be encapsulated effectively inside the lipophilic cores, whereas the hydrophilic shell endow the resulted nanocomposites with excellent biocompatibilities and simplification for further ligand coupling. Magnetic nanoparticles, especially well-dispersed superparamagnetic iron oxide nanoparticles (SPIO NPs), have been clinically applied for magnetic resonance imaging (MRI) contrast enhancement. However, high quality SPIO NPs (e.g. Fe_3_O_4_ nanocrystals) with controlled particle size and better superparamagnetism are usually synthesized via high temperature decomposition of metal complexes in organic phase [4, 5]. Therefore, polymeric micelle encapsulation is considered as a useful surface engineering strategy to obtain SPIO nanocomposites with improved water solubility, biocompatibility and similar magnetic properties to the original SPIO NPs. Moreover, surface coating by amphiphilic polymers is reported as an important factor in modulating the relaxation rate of SPIO-based MRI contrast agent. As reported by previous studies [6–8], clusters of SPIO NPs aggregated inside the hydrophobic core of micelle indicated much better MRI contrast compared with the single SPIO NPs.

To form polymeric micelle structures, amphiphilic comb-like graft copolymers are widely researched, especially those developed from natural polysaccharides, due to their intrinsic biocompatibility [9]. Amongst those widely used polysaccharides, dextran is a microbial origin natural polysaccharide, which has long been investigated as valuable antithrombotic and plasma volume expander in clinical setting and has become of interest as biomaterials. Nanoplatforms developed from amphiphilic dextran have attracted many researchers’ interests as carrier materials because of their excellent biocompatibility, zero net charge and abundant hydroxyl for further chemical coupling reactions. Consequently, dextran micelles were considered as an attractive candidate for SPIO nanoparticles encapsulation to form water-stable colloids for biomedical applications [10, 11]. Thus, it is very interesting to develop amphiphilic dextran and corresponding micelles for SPIO NPs-loading and study its feasibility as MRI contrast agent.

Amphiphilic dextran is typically synthesized by the conjugation of hydrophobic sidechains onto the hydroxyl functionality, leading to ‘grafted’ dextran derivatives [12]. Hydrophobic small molecules such as stearic acid and cholesterol are commonly conjugated through the ‘grafting onto’ approach [10, 13], while synthetic polymers (e.g. polycaprolactone, PCL) can be introduced either by the ‘grafting onto’ protocol or the ‘grafting from’ method using dextran as macroinitiator under high temperature [14]. Generally, several chemical processes including group activation, polymerization reaction and purification process are always involved for the preparation of amphiphilic dextran and corresponding micelle structures. Therefore, this study aims to develop facile protocols for the synthesis of dextran micelle and investigate its performance as vehicle for SPIO NPs encapsulation.

**Figure 1.**
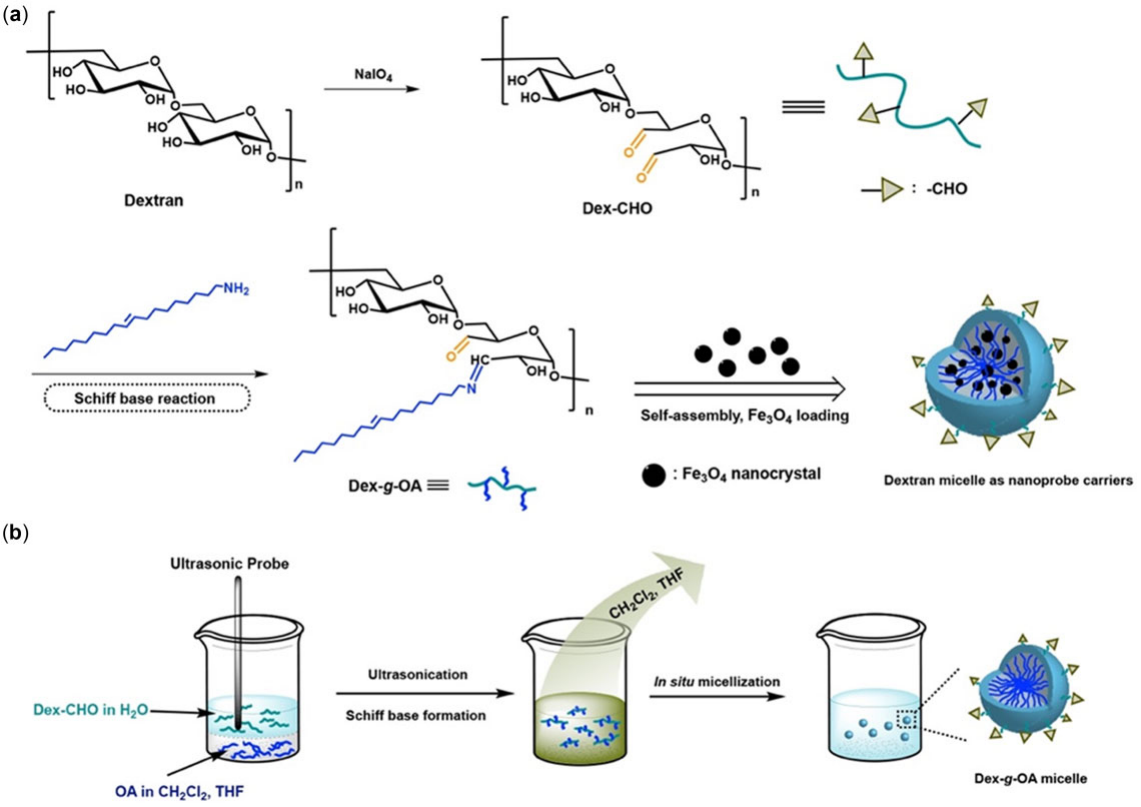
Schematic illustration of the preparation of amphiphilic dextran (Dex-*g*-OA) and Fe_3_O_4_ NPs-loaded dextran micellar nanocomposites (**a**), and the *in situ* self-assembly process (**b**). *Note*: vicinal diols on a glucose moiety may be attacked by periodate ions between both the C2–C3 or C3–C4 in the oxidation process of dextran, and only cleavage at C3–C4 bond is shown in the scheme.

Periodate oxidation, which can convert 1,2-dihydroxyl groups to paired aldehyde groups, has long been used for industrial production of oxidized starch [15, 16]. The oxidized dextran, also known as polyaldehyde dextran, is widely researched as multivalent drug carrier due to the chemical reactivity of aldehyde groups [17, 18]. Typically, the pendent aldehyde groups on the oxidized dextran are available to form Schiff base in presence of primary amines under mild reaction conditions [19, 20], which is demonstrated as an effective protocol to form hydrophobized dextran for surface functionalization of magnetic nanoparticles [21]. On this basis, a facile method for the preparation of amphiphilic dextran derivative was developed herein via the periodate oxidation and Schiff base reaction of dextran. Corresponding micelle nanoparticles were formed by an *in situ* assembly process and investigated for the encapsulation of SPIO NPs. As shown in Fig. 1, polyaldehyde dextran (Dex-CHO) was prepared by periodate oxidation of dextran, and hydrophobic oleylamine (OA) sidechains were conjugated onto the dextran backbones via the ultrasonication-assistant Schiff base reaction. The resulted amphiphilic dextran (Dex-*g*-OA) would self-assemble into nanosized micelles *in situ* in the reaction solution (THF/H_2_O or CH_2_Cl_2_/H_2_O), which was proved and monitored by the fluorescence resonance energy transfer (FRET) technology. The *in situ* self-assembly process was then applied successfully for the encapsulation of hydrophobic iron oxide nanoparticles (Fe_3_O_4_ NPs), leading to a series of superparamagnetic nanocomposites as MRI contrast agents. It is noteworthy that the *in situ* self-assembly protocol may provide a promising methodology for the surface engineering of hydrophobic nanoparticles with low cost and energy consume.

## Materials and methods

### Materials

Dextran T40 (Mw = 40 000 Da) and sodium periodate (NaIO_4_, AR, 99.5%) were purchased from Aladdin CO. Ltd. (Shanghai, China). Sulfo-Cyanine 5 amine (sulfo-Cy5-NH_2_) and Sulfo-Cyanine 5.5 amine (sulfo-Cy5.5-NH_2_) were purchased from Xi’an Ruixi Biological Technology CO. Ltd (Xi’an, China). Iron (III) acetylacetonate (Fe(acac)_2_, AR, 97%), 1,2-hexadecanediol (AR, 90%), benzyl ether (AR, 98%), potassium ferrocyanide (AR, 99%), oleic acid (AR, 70%) and oleylamine (OA, AR, 70%) were purchased from Sigma-Aldrich Corporation. All solvents and reagents were used as received. Dulbecco’s modified Eagle medium and fetal bovine serum were purchased from Invitrogen (California). The human liver cancer cell line HepG2 was obtained from the American Type Culture Collection.

### Synthesis of amphiphilic Dex-*g*-OA micelles

Oxidized dextran (also known as polyaldehyde dextran, Dex-CHO) was prepared by the periodate oxide of dextran with sodium periodate (mole ratio of glucose/${\text{IO}}_{4}^{-}$ at 1:1), and the oxidized degree was determined as 58% by hydroxylamine hydrochloride titration following previous reports [22, 23]. Briefly, 5.0 g of dextran was dissolved in double distilled water (50 ml), NaIO_4_ (6.59 g) was then added into the resulted solution. After stirring for 4 h at room temperature, an equimolar amount of glycerol was dropped into the reaction, and the resulted mixture was dialyzed against deionized water for 3 days, solid Dex-CHO was then obtained after lyophilization. Subsequently, OA molecules were grafted onto the dextran backbone via Schiff base linkages and Dex-*g*-OA micelles were formed as follow: oxidized dextran (25.0 mg) was dissolved in 5 ml of double distilled water, 9.6 mg of the oleylamine in 0.5 ml of THF (or CH_2_Cl_2_) was then added dropwise into the Dex-CHO solution under ultrasonication (70% amplitude, probe ultrasonicator, 1.0 min, SCIENTZ-JY96-IIN, China). The mole ratio of –CHO/–NH_2_ keep constant at 5/1 for all these formulations. The resultant emulsion was shaken at room temperature until THF (or CH_2_Cl_2_) was completely evaporated, leading to the formation of amphiphilic Dex-*g*-OA and *in situ* self-assembly of Dex-*g*-OA micelles. Dynamic light scattering (DLS) size distribution and zeta-potential of the resulted micelles were studied by a Nanosizer (Zetasizer Nano ZS, Malvern, UK). A field emission scanning electron microscope (FE-SEM, FEI Nova Nano SEM 450, USA) was used to observe their size and morphology. All these samples were diluted 10-fold with SPIO concentration at 0.01 mg ml^**−1**^.

In order to confirm its molecular structure, the obtained Dex-*g*-OA polymer was precipitated in ethyl alcohol and washed three times. After being dried in vacuum, the products were characterized by nuclear magnetic resonance (^1^H-NMR, AM400, Bruker, Switzerland) and Bruker Tensor 27 FT-IR spectrometer.

### Critical micelle concentration measurement of the Dex-*g*-OA

Critical micelle concentration (CMC) of the Dex-*g*-OA was determined by fluorescence probe based on pyrene using luminescence spectrometer (F-7500, Hitachi, Japan) [24]. Briefly, Pyrene was dissolved in acetone with low concentration at 6 × 10^−5 ^mol·l^−1^ and quantitatively added into a series of containers, after acetone was evaporated completely, aqueous solution of pre-prepared Dex-*g*-OA micelles with various concentration were added into these containers and shaken for 24 h. Fluorescence spectra of each sample was performed with an optimal emission wavelength at 395 nm, and CMC value of the Dex-*g*-OA was measured from fluorescence intensity ratio between 338 and 334 nm (*I*_338_/*I*_334_).

### Fabrication of Cy@Dex-*g*-OA FRET micelles and FRET ratio calculation

In order to detect the self-assembly of Dex-*g*-OA and formation of micelle structures, Cy5- or Cy5.5-labeled Dex-*g*-OA were synthesized and Cy@Dex-*g*-OA FRET micelles were prepared. Briefly, a solution of sulfo-Cy5-NH_2_ or sulfo-Cy5.5-NH_2_ in water (1.0 mg) was added into an aqueous solution of Dex-CHO while magnetically stirring. The mole ratio of the fluorescent dye to the glucose unit was kept at 1/2000. The resultant solution was stirred for 48 h in dark, and then dialyzed (MWCO: 7000 Da membrane) against deionized water in dark until no fluorescence was detected in the dialysate. The Cy5- or Cy5.5-labled dextran (Cy5@Dex-CHO or Cy5.5@Dex-CHO) were lyophilized and leading to blue or green powder.

Cy@Dex-*g*-OA FRET micelles were then fabricated as follows: Cy5@Dex-CHO (10 mg) and Cy5.5@Dex-CHO (15 mg) was dissolved in 5 ml H_2_O (mole ratio of Cy5/Cy5.5 at 1:1), 9.6 mg of the oleylamine in 0.5 ml of THF (or CH_2_Cl_2_) was then added dropwise into the resulted solution under ultrasonication (70% amplitude, probe ultrasonicator, 1.0 min, SCIENTZ-JY96-IIN, China). The resulted mixture was shaken at room temperature to evaporate the organic solvent (THF or CH_2_Cl_2_), at selected time interval, fluorescence spectra of the mixture was measured using a fluorescence spectrometer (F-4600 FL, Hitachi, Japan) with an excitation wavelength of 640 nm and slit width of 5 nm. Emission spectra were collected from 500 to 800 nm, and the FRET ratio (*r*) was calculated from the intensity of the fluorescence at 680 and 720 nm using the following equation.

$$r=I_{A}/\left(I_{D}+I_{A}\right),$$

where *I*_A_ and *I*_D_ were the fluorescence intensities of Cy5.5 (acceptor) and Cy5 (donor) at 695 nm and 665 nm, respectively.

### Synthesis of Fe_3_O_4_-loaded Dex-*g*-OA micelles

Superparamagnetic Fe_3_O_4_ nanocrystals used in this study were synthesized via the thermal decomposition process of Fe(acac)_3_, following the method developed by Sun *et al*. [4]. Briefly, mixture of Fe(acac)_3_ (2 mmol), 1,2-Hexadecanediol (2 mmol), oleic acid (2 mmol) and oleylamine (2 mmol) in 20 ml benzyl ether was heated to 200°C and maintained for 2 h, and then heated to reflux for 1 h. After cooling to room temperature, the resulted Fe_3_O_4_ nanocrystals were precipitated using ethyl acetate and then dispersed in hexane.

Fe_3_O_4_-loaded Dex-*g*-OA nanocomposites (Fe_3_O_4_@Dex-*g*-OA) were then prepared by similar process for the Dex-*g*-OA micelle formation, with formulation of Dex-CHO/Fe_3_O_4_ (mass) = 3/1 (M1), 5/1 (M2), 7/1 (M3), 10/1 (M4) and 100/1 (M5). Briefly, Fe_3_O_4_ NPs (1.0 mg) and OA (the amount of OA was calculated with –CHO/–NH_2_ mole ratio at 5/1) was dispersed in 1.0 ml THF, the resulted mixture was dropped into 10 ml of aqueous solution of Dex-CHO (3.0, 5.0, 7.0, 10.0 or 100 mg) under ultrasonication (70% amplitude, 1 min, probe ultrasonicator, SCIENTZ-JY96-IIN, China). Fe_3_O_4_@Dex-*g*-OA nanocomposites in water were obtained after evaporation of THF. Morphology and particle size of the Fe_3_O_4_-loaded nanocomposites were studied with FE-SEM, transmission electron microscopy (HRTEM, FEI, Tecnai G2 F30 S-Twin, 300 kV) and DLS measurement. Energy disperse spectroscopy (EDS) elemental distribution was monitored by an Octane Pro energy dispersive X-ray spectrometer.

### *In vitro* cytotoxicity and cell labeling study

Firstly, the cytotoxicity of Fe_3_O_4_@Dex-*g*-OA nanocomposites against HepG2 cells was investigated by the Cell Counting Kit-8 (CCK-8) method. Briefly, HepG2 cells were seeded in 96-well palates at density of 5 × 10^3^ cells per well. After cultured for 24 h at 37°C, 5% CO_2_ incubator, the medium was removed and replaced with fresh medium containing Fe_3_O_4_@Dex-*g*-OA (M1, M2, M3, M4 and M5) to make a final Fe concentration of 10 μg ml^−1^ and different Fe concentration (4, 6, 8, 10, 12 μg ml^−1^) of the M3. After another 24 h incubation, the medium was removed, and the cells were washed with PBS. Then, 200 μl of medium containing 10% CCK-8 reagent was added to each well followed by 6 h of incubation at 37°C, 5% CO_2_ incubator. Cell viability was determined by measuring the absorbance of each well at 450 nm using a Max M5 microplate reader (Molecular Devices, CA, USA). All experiments were conducted in quadruplicate.

Then, HepG2 cells were seeded on six-well tissue culture palates at 2 × 10^5^ cells per well and cultured for 24 h before labeling. The medium was removed from each well and cells were washed twice with PBS, and the Fe_3_O_4_@Dex-*g*-OA nanocomposite (M1, M2 and M3) in medium were added to the HepG2, leading to a final concentration of iron at 10 μg ml^−1^. The cell cultures were kept for 24 h at 37°C, 5% CO_2_ incubator. The medium was then removed, and cells were washed two times with PBS. The Fe_3_O_4_@Dex-*g*-OA labeling efficiency of HepG2 was examined with Prussian blue staining. Briefly, the labeled cells were fixed with 4% paraformaldehyde, followed by incubation at room temperature for 30 min. The cells were then washed with PBS and incubated for 4 h with 5% potassium ferrocyanide in 10% hydrochloric acid. Finally, cells were washed again with PBS and observed using an optical microscope.

### Magnetization and *T*_2_ relaxivity studies

Magnetic property of the Fe_3_O_4_@Dex-*g*-OA nanocomposites in aqueous phase were studied using an MPMS3 Quantum Design SQUID magnetometer (Quantum Design Inc., USA) at 300 K with the scope of −30 to 30 kOe, four quadrants. Solid content of the samples was measured after lyophilization.

*T*_2_ relaxivity of the Fe_3_O_4_@Dex-*g*-OA nanocomposites was measured at 0.5 T on an NMR relaximetry (PQ001-20-015V, Shanghai Niumag company LTD, China). The transverse relaxation times (*T*_2_) of a series of aqueous solution of the Fe_3_O_4_@Dex-*g*-OA nanocomposites with different Fe concentrations were measured. Relaxivity (*r*_2_) value was calculated through the curve fitting of 1/*T*_2_ relaxation time (s^−1^) vs the iron concentration (mM).

## Results and discussions

### *In situ* formation of Dex-*g*-OA micelles

**Figure 2.**
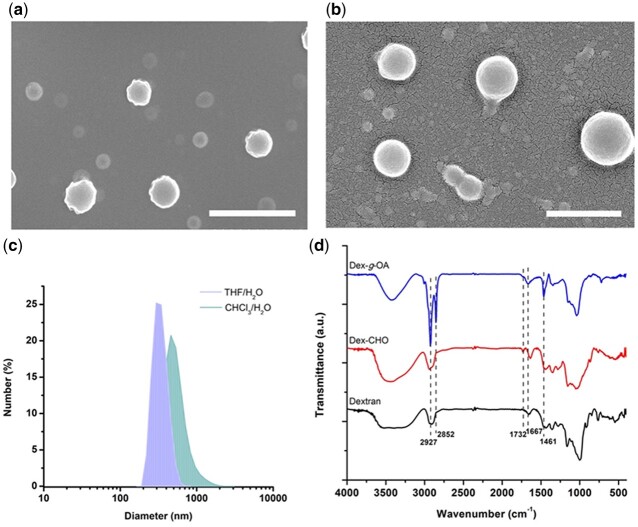
SEM images of Dex-*g*-OA micelles formed *in situ* in the reaction solution (**a**) THF/H_2_O, (**b**) CH_2_Cl_2_/H_2_O (scale bar: 2 µm); (**c**) DLS size distribution of the micelles; (**d**) FT-IR spectra of the dextran, Dex-CHO and Dex-*g*-OA synthesized in THF/H_2_O system.

Amphiphilic Dex-*g*-OA were prepared conveniently by Schiff base reactions between oleylamine (OA) and polyaldehyde dextran (Dex-CHO) in aqueous solution at room temperature, and corresponding micelles were ‘one-pot’ formed *in situ* in the reaction system, as shown in Fig. 1b. SEM study of the resulted aqueous mixture shown in Fig. 2 indicated that nanosized Dex-*g*-OA micelles were successfully formed both in the THF/H_2_O (Fig. 2a) and CH_2_Cl_2_/H_2_O (Fig. 2b) systems. DLS measurements in Fig. 2c showed that the mean diameters of the Dex-*g*-OA micelles were 338.1 and 549.7 nm, respectively. Both the SEM and DLS demonstrated that amphiphilic dextran derivative (Dex-*g*-OA) were successfully formed and self-assembled into nano-scale micelles *in situ* by the ‘one-pot’ process. However, the complicated components and combination of several processes (e.g. formation of Dex-*g*-OA polymer and self-assembly of Dex-*g*-OA may occur simultaneously) in the reaction system may lead to the broad particle size distribution, which was described as ‘the more components are present in the solution, the more complex is the self-assembly process’ by Eisenberg *et al*. [25].

In addition, CMC measurement via the fluorescence spectra (Supplementary Fig. S1) showed that both the obtained Dex-*g*-OA have CMC value at the same order of magnitude of 10^−2 ^mg ml^−1^. Interestingly, the CMC value of Dex-*g*-OA prepared in CH_2_Cl_2_/H_2_O (6.8^ ^×^ ^10^−2 ^mg ml^−1^) is slightly higher than that formed in THF/H_2_O (3.9^ ^×^ ^10^−2 ^mg ml^−1^), indicating that the corresponding Dex-*g*-OA micelles assembled in THF/H_2_O solution is more stable. The phenomenon may be caused by a different chain stretching behavior (solubility parameter) of OA molecule in the common solvent (CH_2_Cl_2_ or THF) [25, 26], which may also lead to the larger particle size of Dex-*g*-OA micelles prepared in CH_2_Cl_2_/H_2_O system. Hence, Dex-*g*-OA micelles formed in THF/H_2_O system were applied in the following study.

To further prove the structure of amphiphilic dextran (Dex-*g*-OA), FT-IR and ^1^H NMR analysis of the resulted products were then carried out. As shown in Fig. 2d, absorption peaks at 1732 cm^−1^ corresponding to the stretching vibration of carbonyl from an aldehyde group (–C=O in –CHO groups) on the spectrum of Dex-CHO was strongly weakened after the grafting of OA molecules, leading to the Schiff base bonds (C=N) as junctions between the dextran backbones and the hydrophobic OA sidechains. Therefore, characteristic band corresponding to the stretching of –C=N– and –C=C– bonds (1690–1620 cm^−1^) was observed in the spectra of Dex-*g*-OA at 1667 cm^−1^, which may be slightly overlapped with the vibration band of water molecule at about 1640 cm^−1^. Moreover, absorption peaks at 1461, 2852 and 2927 cm^−1^ corresponding to the –CH_2_– and –CH_3_ bonds on the OA chains were clearly observed in the spectrum of Dex-*g*-OA, indicating the successful formation of Dex-*g*-OA.

**Figure 3.**
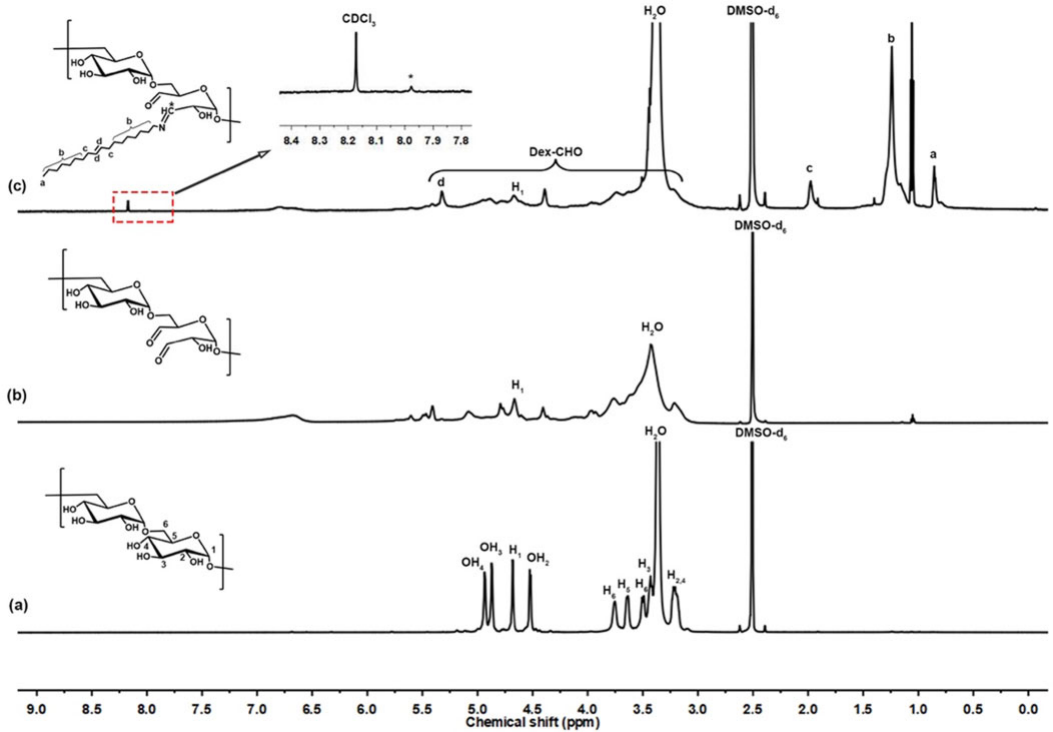
^1^H NMR spectra of the dextran (DMSO-d_6_) (**a**), Dex-CHO (DMSO-d_6_) (**b**) and Dex-*g*-OA (synthesized in THF/H_2_O, CDCl_3_/DMSO-d_6(v:v)_ = 1/5) (**c**).

The amphiphilic Dex-*g*-OA polymer prepared by the ‘one-pot’ method in THF/H_2_O was also characterized by NMR spectroscopy, and the pure dextran T40 and Dex-CHO were both measured for comparison. As shown in Fig. 3a, peaks in the ^1^H NMR spectrum of dextran between *δ* 3.0 and 4.0 are attributed to protons at positions 2, 3, 4, 5 and 6, while chemical shift for the anomeric proton (H_1_) and protons from hydroxy groups (OH_2,3,4_) at the glucose unit are found between *δ* 4.3 and 5.1. After the periodate oxidation, distinctive peaks between *δ* 3.0 and 6.0 are observed obviously in the spectrum of Dex-CHO (Fig. 3b), which are likely assigned to protons at the glucose residues and oxidized residues according to previous literatures [27]. However, detailed assignment of those chemical shifts is complicated due to the formations of intra or inter-chain hemiacetals from the aldehyde groups and nearby hydroxyl groups [28, 29]. Moreover, the vicinal diols on glucose unit can be attacked by periodate ions both between C_3_–C_4_ or C_2_–C_3_ in the oxidation process, and even second oxidation would happen along with releasing of formic acid. According to Ishak and Painter [28], the cleavage at C_3_–C_4_ bond is much more favored comparatively to the cleavage at C_2_–C_3_ bond, therefore, Dex-CHO with C_3_–C_4_ cleavage bond is represented in the scheme. In the spectrum of Dex-*g*-OA (Fig. 3c), chemical shift of the grafted OA sidechains was showed as below: *δ* 0.83 (–CH=N–(CH_2_)_7_CH_2_CH=CHCH_2_(CH_2_)_6_C**H**_3_, a, 3H), 1.23 (–CH=N–(C**H**_2_)_7_CH_2_CH=CHCH_2_(C**H**_2_)_6_CH_3_, b, 26H), 1.75 (–CH=N–(CH_2_)_7_C**H**_2_CH=CHC**H**_2_(CH_2_)_6_CH_3_, c, 4H), 5.35 (–CH=N–(CH_2_)_7_CH_2_C**H**=C**H**CH_2_(CH_2_)_6_CH_3_, a, 2H), and a weak peak corresponding to the chemical shift for the proton on Schiff base (–C**H**=N–, *, 1H) was observed at *δ* 7.98, indicating the successful graft of OA onto the backbone of Dex-CHO via Schiff base formation. Moreover, the substitution degree (SD) of OA calculated from the ^1^H NMR spectrum of Dex-*g*-OA was about 18%, which means there are approximate 18 OA chains grafted onto 100 of the residue saccharide units, and similar OA SD was determined from the Dex-*g*-OA obtained in CH_2_Cl_2_/H_2_O (Supplementary Table S1 and Fig. S2), According to the OA SD, the theoretical average molecular weight of amphiphilic dextran units is about 205.2 Da.

### FRET study of Dex-*g*-OA micelle

**Figure 4.**
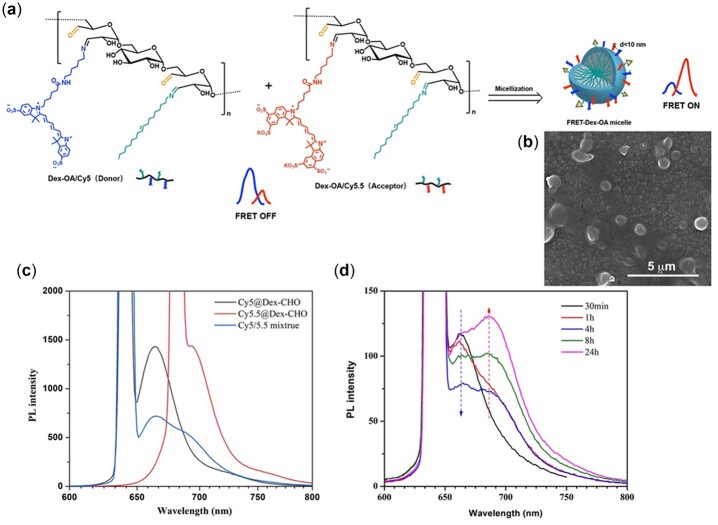
FRET study of the self-assembly behavior of Dex-*g*-OA. Schemetic illustration (**a**) and SEM image (**b**) of Dex-*g*-OA FRET micelles; fluorescence spectra of the fluorophores pair (Cy5/Cy5.5) (**c**) and the reaction solution of THF/H_2_O (**d**). (ex of Cy5@Dex-CHO and Cy5/5.5 mixtrue: 640 nm; ex of Cy5.5@Dex-CHO: 680 nm).

FRET-based technology has long been considered as an effective tool for investigating the structure, drug/nanoparticle loading, stimuli-responses and release kinetics of polymeric micelles [30–32]. Notably, the self-assembling behaviors of amphiphilic polymers can be assessed by FRET probes labeling and detection of their energy transfer signal along with the distance changes between the two fluorophores. In this study, the extensively used near-infrared dyes, water soluble Cy5 (Sulfo-Cy5-NH_2_) and Cy5.5 (Sulfo-Cy5.5-NH_2_) were used as an FRET pair, and conjugated separately onto the backbone of Dex-CHO. After grafting of OA sidechains, a pair of Cy-labeled amphiphilic dextran (Cy5@Dex-*g*-OA and Cy5.5@Dex-*g*-OA) were formed and self-assembled into Cy@Dex-*g*-OA micelles *in situ* in the reaction systems, as shown in Fig. 4a, the FRET phenomenon would be observed if the fluorophores pair (Cy5/Cy5.5) are close to each other (*d* < 10 nm). In addition, the ^1^H NMR spectroscopy study (Supplementary Fig. S3) of the resulted products demonstrated the successful conjugation of OA molecules.

For comparison, fluorescence spectra of both the Cy5 and Cy5.5 labeled Dex-CHO was studied first and shown in Fig. 4c. The Cy5 labeled Dex-CHO (Cy5@Dex-CHO, donor) had strong fluorescence emission (Em) peak at 665 nm with an excitation wavelength (Ex) of 640 nm, while Dex-CHO labeled with Cy5.5 (Cy5.5@Dex-CHO, acceptor) showed a distinct emitted wavelength at 695 nm under exciting at 680 nm. In addition, no FRET phenomenon was detected from the mixture of Cy5@Dex-CHO and Cy5.5@Dex-CHO before reacting with OA sidechains. After grafting hydrophobic OA molecules, FRET signals of the reaction mixture were detected at selected time interval. As shown in Fig. 4d, a time-dependent fluorescence spectra of the reaction mixture (THF/H_2_O system) were observed along with the formation of Dex-*g*-OA FRET micelles. Once excited at 640 nm, the strong Cy5 fluorescence at 665 nm weakened gradually after 1 h, and a shoulder peak of Cy5.5 at 690 nm appeared meanwhile and enhanced dramatically after 4 h. At 24 h, a strong Cy5.5 fluorescence and weak Cy5 fluorescence of the reaction solution was observed, indicating that amphiphilic dextran formed via Schiff base reactions assembled into micelles and a strong FRET occurred. Similar FRET phenomenon was observed in the reaction system of CH_2_Cl_2_/H_2_O (Supplementary Fig. S4). The FRET ratio (*r*) at different time intervals was calculated and listed in Supplementary Table S2. At the preliminary stage of the reaction, few amphiphilic polymers and corresponding micelle were formed, the calculated FRET ratio was 0.27 at 0.5 h. Once the Cy@Dex-*g*-OA FRET micelle formed *in situ* in the mixture, the FRET ratio increased from 0.38 at 1 h to 0.52 at 24 h. After evaporation of THF, nanosized micelle structures (Cy@Dex-*g*-OA) were clearly visible under SEM studies (Fig. 4b).

### *In situ* SPIO loading

To investigate their performance as carrier systems, the *in situ* self-assembled Dex-*g*-OA micelles developed in this study were applied for iron oxide (Fe_3_O_4_) NPs loading. Since superparamagnetic Fe_3_O_4_ nanocrystal is a widely used MRI contrast agent, the resulted Fe_3_O_4_/micelle nanocomposite may find potential applications in biomedical imaging.

**Figure 5.**
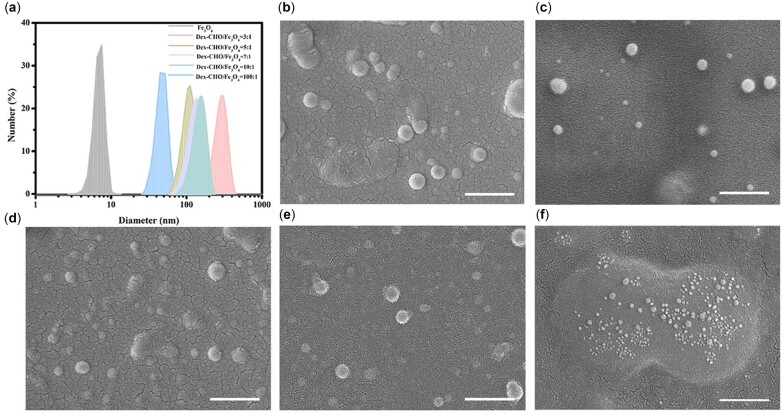
DLS (**a**) and SEM images of Fe_3_O_4_@Dex-*g*-OA nanocomposites prepared with Dex-CHO/Fe_3_O_4_ mass ratio of 3/1 (**b**), 5/1 (**c**), 7/1 (**d**), 10/1 (**e**) and 100/1 (**f**) (scale bar: 500 nm).

High-quality Fe_3_O_4_ nanocrystals are usually synthesized in organic phase at high temperatures for better control of particle size and morphology [4, 33], DLS (Fig. 5a) study indicated that Fe_3_O_4_ nanocrystals with narrow particle size distribution in hexane (7.1 ± 1.1 nm) were synthesized by thermal decomposition of Fe(acac)_3_. The crystal diameter measured by selecting different representative areas under TEM was 5.6 ± 1.0 nm (30 particles), and lattice fringes were clearly visible in the high-resolution TEM (HRTEM) images. Fe_3_O_4_ nanoparticles were then successfully encapsulated into the hydrophobic core of Dex-*g*-OA micelles via the *in situ* process, leading to Fe_3_O_4_@Dex-*g*-OA nanocomposites well dispersed in water. Five different formulations with various mass ratio of Dex-CHO/Fe_3_O_4_ were tried for the phase transfer of Fe_3_O_4_ nanoparticles, SEM study (Fig. 5b–f) showed that all of these samples can form spherical nanoparticles in the reaction solutions, and Fe_3_O_4_@Dex-*g*-OA nanocomposites prepared with Dex-CHO/Fe_3_O_4_ mass ratio of 5/1, 7/1 and 10/1 showed higher stability. However, clearly visible sediment was observed after about 24 h in the solution prepared with Dex-CHO/Fe_3_O_4_ mass ratio of 3/1, which may be caused by the aggregation of Fe_3_O_4_@Dex-*g*-OA nanoparticles, as shown in the SEM study and Zeta potential study (Supplementary Fig. S5 and Table S3). In addition, nanoparticles with relatively smaller diameter were clearly observed in the SEM image (Fig. 5f) of sample prepared with Dex-CHO/Fe_3_O_4_ mass ratio of 100/1, indicating that the excess Dex-*g*-OA molecules may lead to numerous Fe_3_O_4_@Dex-*g*-OA nanocomposites with several Fe_3_O_4_ nanocrystal inside their micelle cores, which was proved by TEM study in Supplementary Fig. S6. EDS analysis (Supplementary Fig. S7) was used for further investigation of the elemental compositions of Fe_3_O_4_@Dex-*g*-OA nanocomposites prepared with Dex-CHO/Fe_3_O_4_ mass ratio of 5/1 (Supplementary Fig. S7a) and 7/1 (Supplementary Fig. S7b). Signals corresponding to the C, O and N element in the Dex-*g*-OA molecules, and Fe element from Fe_3_O_4_ NPs were identified.

**Figure 6.**
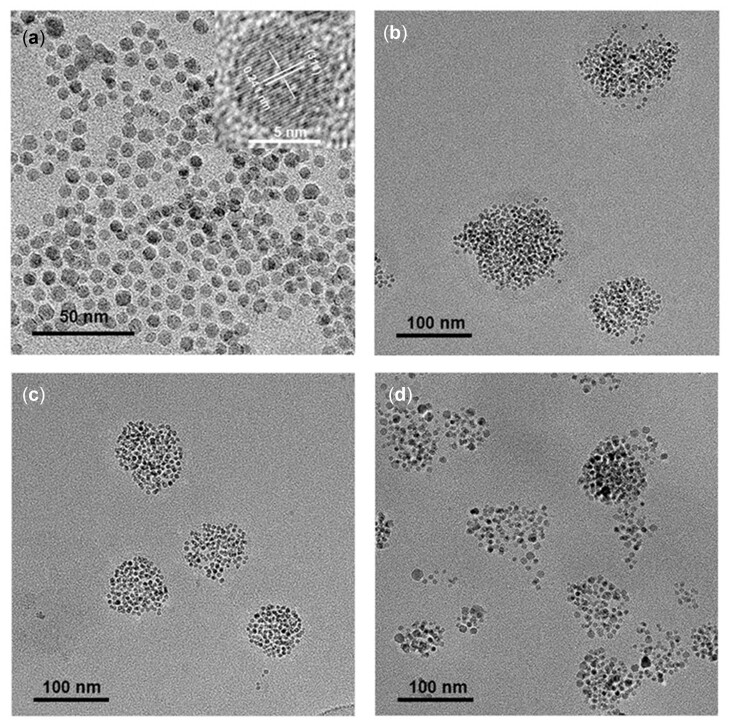
TEM images of Fe_3_O_4_ nanocrystals (**a**) and Fe_3_O_4_@Dex-*g*-OA nanocomposites prepared with Dex-CHO/Fe_3_O_4_ mass ratio of 5/1 (**b**), 7/1 (**c**) and 10/1 (**d**).

Particle size distribution of the fresh prepared nanocomposites in aqueous solution was measured by DLS and is shown in Fig. 5a, and the corresponding average particle size (hydrodynamic diameter by number) was calculated. As shown in Table 1, particle size of the Fe_3_O_4_@Dex-*g*-OA nanocomposite is significantly larger than that of Fe_3_O_4_ particles, which was caused by the Dex-*g*-OA coating and multiple loaded Fe_3_O_4_ nanocrystals. TEM measurement of the resulted Fe_3_O_4_@Dex-*g*-OA nanocomposite in Fig. 6 demonstrated that Fe_3_O_4_ particles were wrapped inside the micelles and packed densely as isolated clusters of multiple nanocrystals. Similar clustering of Fe_3_O_4_ nanocrystals encapsulated in polymeric micelles were commonly reported by previous publications, and particle size of the nanocomposites would increase with lower ratio of polymer/Fe_3_O_4_, since more Fe_3_O_4_ particles are assembled into clusters with less amount of amphiphilic polymers [34]. Interestingly, the DLS particles size of Fe_3_O_4_@Dex-*g*-OA nanocomposites formed in this study with formulation M2, M3 and M4 (Dex-CHO/Fe_3_O_4_ ratio: 5/1, 7/1 and 10/1) indicated a different profile by a larger particle diameter with increased polymer/Fe_3_O_4_ ratio. Since Fe_3_O_4_ nanocrystals synthesized via thermal decomposition are covered with hydrophobic aliphatic chains from oleic acid and oleylamine, and those oleylamine molecules on the surface of Fe_3_O_4_ nanocrystals would react with Dex-CHO during the *in situ* Fe_3_O_4_ loading process, Dex-*g*-OA with different OA grafting ratio (as shown in Table 1, higher than 18% determined by ^1^H NMR study of the pure Dex-*g*-OA micelle) would form in the resulted Fe_3_O_4_@Dex-*g*-OA nanocomposites. Dex-*g*-OA with higher SD of OA side chains would self-assembled into polymeric micelles with smaller particle size, due to a stronger hydrophobic interaction between their aliphatic side chains, which was commonly approved by previous publications focus on micelle structures formed by amphiphilic polysaccharides [12, 35, 36]. Therefore, Fe_3_O_4_@Dex-*g*-OA nanocomposites prepared with formulation M4 showed a relatively larger diameter at 116.2 ± 53.7 nm, and particle size of Fe_3_O_4_-loaded Dex-*g*-OA micelle decreased obviously compared with that of Dex-*g*-OA micelle without Fe_3_O_4_ loading.

**Table 1.** DLS size, magnetization and *T*_2_ relaxivities of the Fe_3_O_4_@Dex-*g*-OA nanocomposites with different formulations

Entry	Dex-CHO/Fe_3_O_4_ (m/m)	SD^a^ of OA	Size (nm)	Magnetization (emu/ml sample)	*T*_2_ relaxivity (${\text{mM}}_{\text{Fe}}^{-1}$·s^−1^)
M1	3/1	29%	188.7 ± 38.0	1.97	139.2
M2	5/1	27%	93.4 ± 47.0	1.79	223.8
M3	7/1	25%	114.3 ± 26.7	1.31	285.2
M4	10/1	24%	116.2 ± 53.7	1.06	327.9
M5	100/1	23%	81.1 ± 30.0	-	-

a Theoretical substitution degree of OA determined by feeding ratio.

### Cytotoxicity and cell labeling

Cytotoxicity of the Fe_3_O_4_@Dex-*g*-OA nanocomposites against HepG2 cells was evaluated by CCK-8 assay after incubation for 24 h (Supplementary Fig. S8). As shown in Supplementary Fig. S8a, cells treated with M1, M2, M3 and M4 showed no obvious cytotoxicity at the Fe concentration of 10 μg/ml. At the same Fe concentration, however, Fe_3_O_4_@Dex-*g*-OA nanocomposite M5 indicated relatively higher cytotoxicity due to a higher Dex-*g*-OA content. A possible reason for the toxic effects of Dex-*g*-OA at high concentration is the high content of aldehyde (–CHO), which are able to interact with electron-rich biological macromolecules and induce general toxicity [17]. In addition, CCK-8 results of M3 in Supplementary Fig. S8b indicated that no significant differences were found in the cellular proliferation between five concentrations. Thus, the Fe_3_O_4_@Dex-*g*-OA nanocomposite M1, M2, M3 and M4 showed no obvious cytotoxicity on the tested cell line.

**Figure 7.**
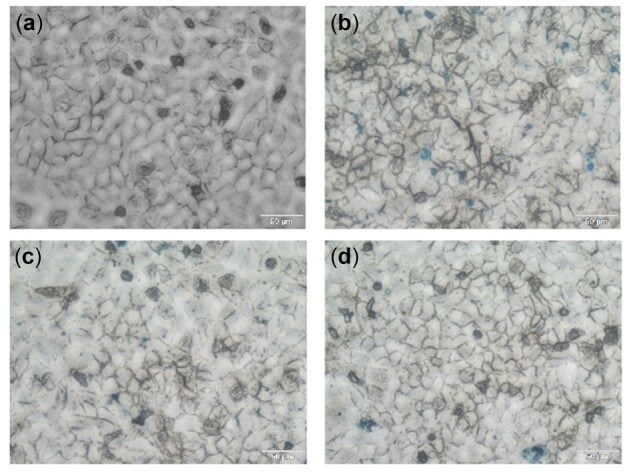
Optical microscopy images of HepG2 cells: without labeling (**a**), Prussian blue stains after labeling with Fe_3_O_4_@Dex-*g*-OA nanocomposites M1 (**b**), M2 (**c**) and M3 (**d**).

Cells were then labeled by simply adding Fe_3_O_4_-loaded nanocomposites (M1, M2 and M3) to the culture medium at Fe concentrations of 10 μg ml^−1^ with incubation time of 24 h. Compared with the blank control in Fig. 7a, HepG2 cells co-cultured with nanocomposite M1, M2 and M3 maintained normal flat and elongated shape, confirming the cytocompatibility of Fe_3_O_4_@Dex-*g*-OA nanocomposites. Moreover, Prussian blue staining of the formulation M1, M2 and M3 labeled cells show that M2 and M3 are mostly located in the cytoplasm and only a few on the cell surface (Fig. 7c and d), indicating an effective cell uptake of the tested nanocomposites. However, for M1, aggregates were mostly attached to the cell surface and were present in the background (Fig. 7b), and cannot be removed through washing, due to the instability, easy agglomeration and large micelle size of M1.

### Magnetization and *T*_2_ relaxivity

Iron oxide nanoparticles with particle size smaller than 20 nm are known to exhibit unique superparamagnetic properties and have long been researched as MRI contrast agents, which are strong enhancers of proton relaxation with superior MR transvers relaxation (*T*_2_) shortening effects [37, 38]. Interestingly, clusters of multiple SPIO nanoparticles were reported to show similar magnetic properties, and much stronger *T*_2_ effects were identified compared with single SPIO particle [6, 39]. Therefore, the magnetic properties of Fe_3_O_4_@Dex-*g*-OA nanocomposites developed in this study were measured and their functions as MRI contrast agent were evaluated.

**Figure 8.**
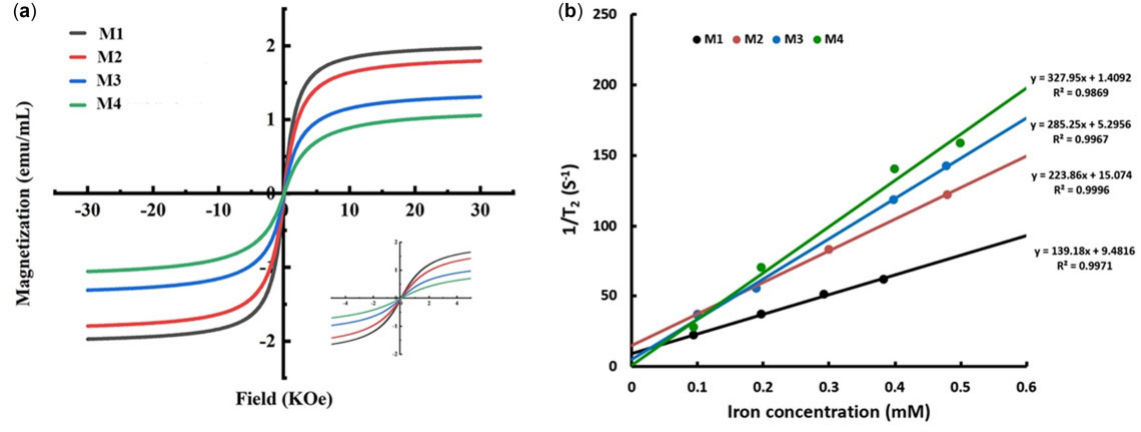
(**a**) Hysteresis loops measured at 300 K (inset shows a zoomed-in plot between −5 and 5 kOe magnetic field) and (**b**) *T*_2_ relaxation rate (1/*T*_2_, s^−1^) as a function of Fe concentration (mM) for the Fe_3_O_4_@Dex-*g*-OA nanocomposites.

The field-dependent magnetizations of Fe_3_O_4_@Dex-*g*-OA nanocomposites in aqueous solution were measured and shown in Fig. 8a, which indicated that the net magnetization of all these samples returned to zero in the absence of an external magnetic field. Typical superparamagnetism of the Fe_3_O_4_@Dex-*g*-OA nanocomposites with formulations M1, M2, M3 and M4 were identified by the magnetization measurement. The saturation magnetization (*M*_s_) was detected under a large external magnetic field while the Fe_3_O_4_ nanocrystals align with the field direction. The *M*_s_ values decreased obviously from 1.97, 1.79, 1.31 to 1.06 emu ml^−1^ of samples with the Dex-CHO/Fe_3_O_4_ mass ratio increased from 3/1 to 10/1. According to previous publications [40–42], the non-magnetic materials coating of iron oxide nanocrystals may result in the decrease of *M*s values, which were supposed as magnetically ineffective layers on the particle surface. Similarly, the higher content of amphiphilic polymers (Dex-g-OA) in the Fe_3_O_4_@Dex-*g*-OA nanocomposites may lead to more magnetically ineffective layer on their particle surface, and hence a decreased *M*s value.

To understand how these Fe_3_O_4_@Dex-*g*-OA nanocomposites performed as MRI contrast agents, the *T*_2_ relaxivities (*r*_2_) of formulation M1, M2, M3 and M4 were measured at 0.5 T using an NMR relaximetry. The *T*_2_ relaxivity represents their net effectiveness of shortening the *T*_2_ (spin-spin) relaxation time and is usually determined by the slope of the linear plot of 1/*T*_2_ against Fe concentration, with units of ${\text{mM}}_{\text{Fe}}^{-1}$·s^−1^. Figure 8b displayed the *T*_2_ relaxivity of the Fe_3_O_4_@Dex-*g*-OA nanocomposites, which indicated that nanocomposites M2, M3 and M4 have relatively higher *r*_2_ value of 223.8, 285.2 and 327.9 ${\text{mM}}_{\text{Fe}}^{-1}$·s^−1^, respectively. Moreover, the *T*_2_ relaxivity increased dramatically with the increasing particle size as shown in Table 1. It is assumed by Josephson *et al.* [43] that the enhancement of spin-spin relaxation indicates the ability of magnetic particles to distort the local magnetic field. Due to their larger magnetic moments per particle in solution, clusters of multiple SPIO nanocrystals with larger diameter would distort the magnetic field in larger volumes of solvent. Similar phenomena were reported for SPIO-loaded mPEG-*b*-PCL and alkylated polyethylenimine micelles [37, 44]. However, a relatively lower *r*_2_ value (139.2 ${\text{mM}}_{\text{Fe}}^{-1}$·s^−1^) of nanocomposite M1 was detected, which may be caused by the aggregations and precipitation of Fe_3_O_4_@Dex-*g*-OA particles with lower stability during the testing process.

## Conclusions

An *in situ* self-assembly method was developed for the facile preparation of amphiphilic dextran micelles, based on the Schiff base reactions between oxidized dextran and oleylamine in aqueous solutions. The self-assembling behavior of the amphiphilic dextran derivative was identified using FRET technique. Hydrophobic SPIO NPs were then successfully encapsulated into the dextran micelles via the *in situ* self-assembly process, leading to a series of superparamagnetic nanocomposites with good biocompatibility and strongly enhanced *T*_2_ relaxivity. Notably, the residual aldehyde groups on the dextran micelle are available for covalent conjugations of functional ligands and allow facile design of multifunctional nanoplatforms in biomedical imaging.

## Supplementary Material

rbac096_Supplementary_Data
