# Supplementary material for: In situ self-assembly of amphiphilic dextran micelles and superparamagnetic iron oxide nanoparticle-loading as magnetic resonance imaging contrast agents
Source: Regen Biomater. 2022 Dec 5;10:rbac096. doi: 10.1093/rb/rbac096 (PMC9847518; doi:10.1093/rb/rbac096)
Supplement: rbac096_Supplementary_Data [file rbac096_supplementary_data.docx]

**Supplementary material**

***In situ* self-assembly of amphiphilic dextran micelles and superparamagnetic iron oxide nanoparticle-loading as magnetic resonance imaging contrast agents**

Linrui Jiang ^a^, Ni Zeng ^a^, Rong Zheng ^a^, Changqiang Wu ^b^, Hongying Su^a,^ *

^a^ Faculty of Chemical Engineering, Kunming University of Science and Technology, 727 South Jingming Road, Kunming, 650500, China

^b^ Sichuan Key Laboratory of Medical Imaging, North Sichuan Medical College, Nanchong, 234 Fujiang Road, Nanchong 637000, China

^*^Corresponding author: hongyingsu@kmust.edu.cn


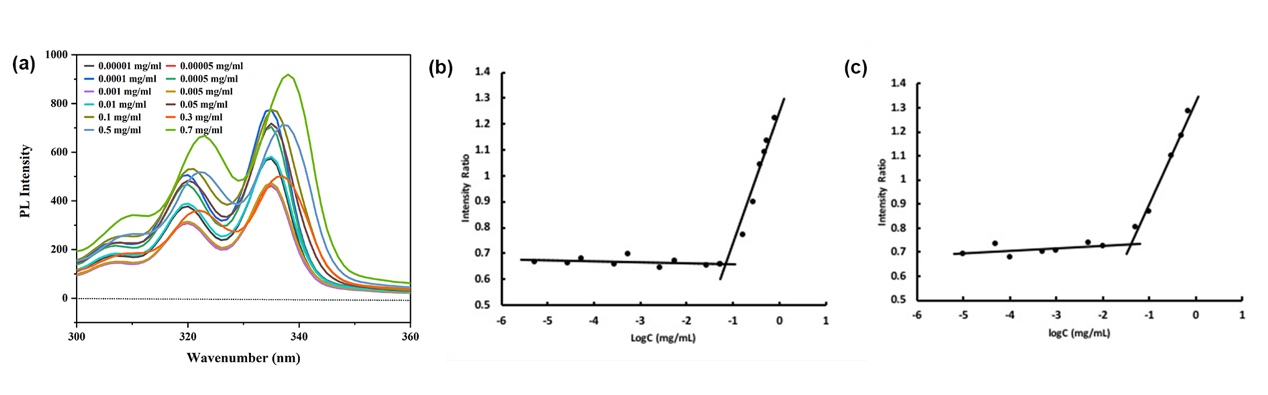


**Fig. S1.** (a) Fluorescence spectra of pyrene solutions with various concentration of Dex-*g*-OA micelles formed in CH_2_Cl_2_/H_2_O; Plot of *I*_338_/*I*_334_ as a function of Dex-*g*-OA micelles concentrations prepared in CH_2_Cl_2_/H_2_O (b) and THF/H_2_O (c).

**Table S1.** Effect of the common solvent on the properties of Dex-*g*-OA micelles.

| Trial | Solvent | SD^a^ (%) | SD^b^ (%) | Size (DLS, nm) | CMC (mg/mL) |
| --- | --- | --- | --- | --- | --- |
| 1 | THF/H_2_O_(v:v)_=1/10 | 23% | 18% | 338.1 ± 74.6 | 3.9 x 10^-2^ |
| 2 | CH_2_Cl_2_/H_2_O_(v:v)_=1/10 | 23% | 19% | 549.7 ± 205.4 | 6.8 x 10^-2^ |

^a^ Theoretical substitution degree of OA determined by feeding ratio.

^b^ Substitution degree of OA determined by ^1^H NMR data.

**
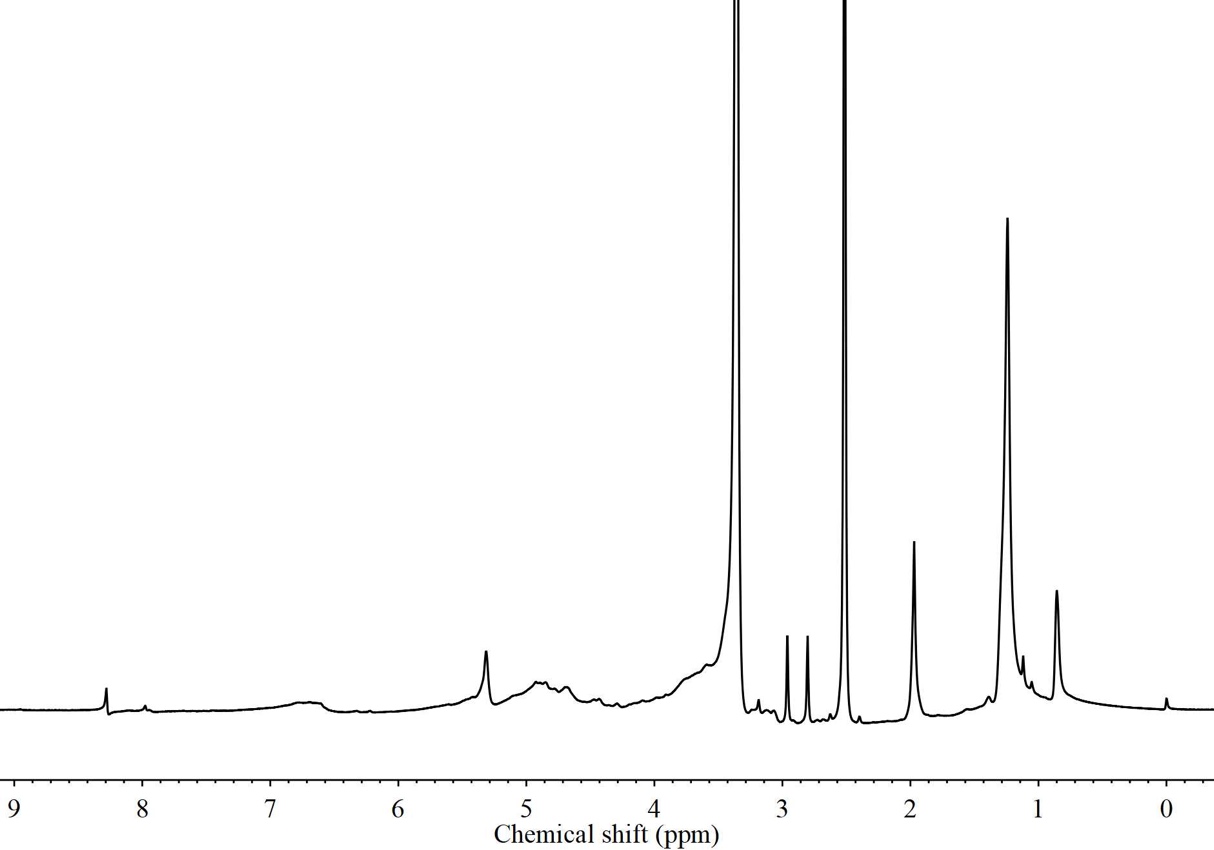
**

**Fig. S2.** ^1^H NMR spectrum of the Dex-*g*-OA synthesized in CH_2_Cl_2_/H_2_O (CDCl_3_/DMSO-d_6(v:v)_=1/5).


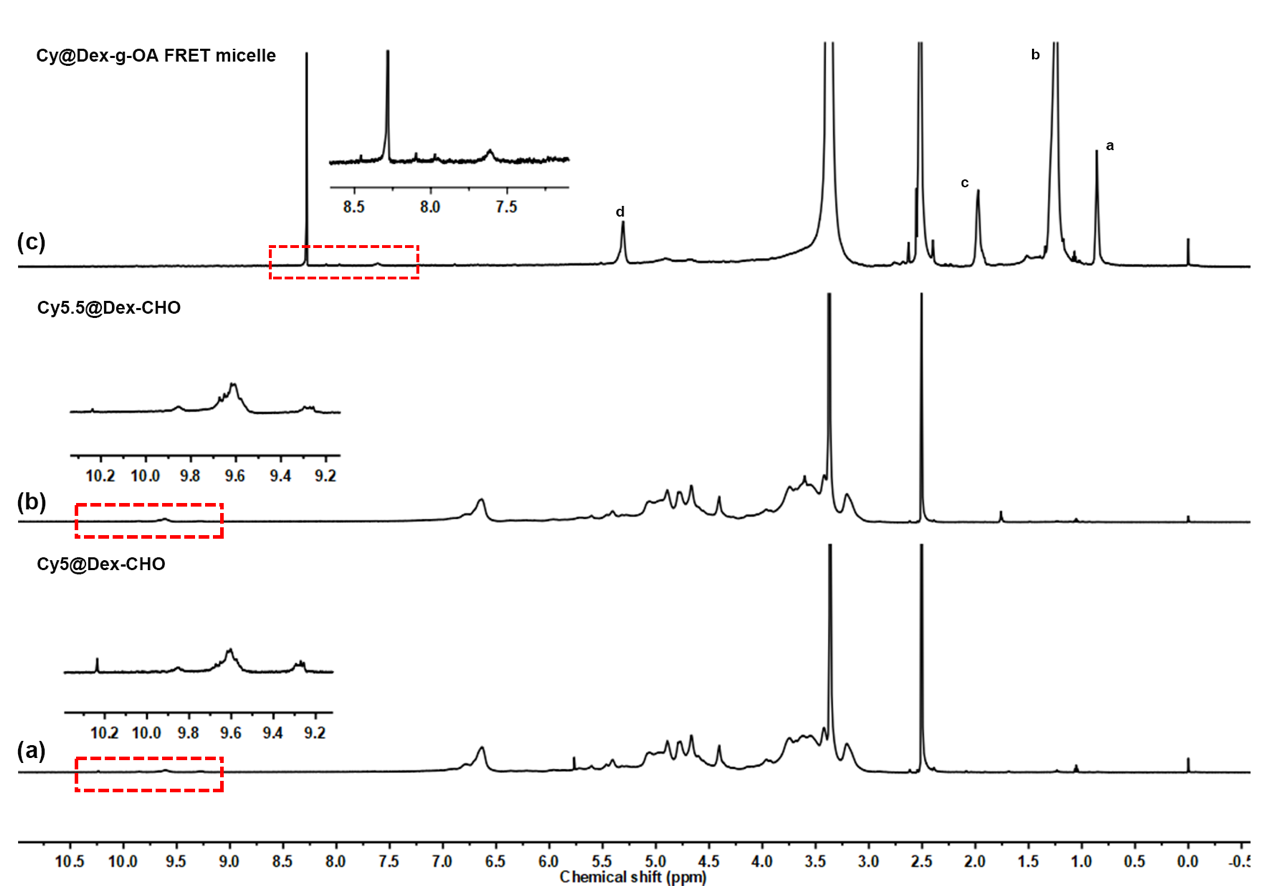


**Fig. S3.** ^1^H NMR spectrum of the Cy5@Dex-CHO, Cy5.5@Dex-CHO and Cy@Dex-*g*-OA micelles synthesized in THF/H_2_O.


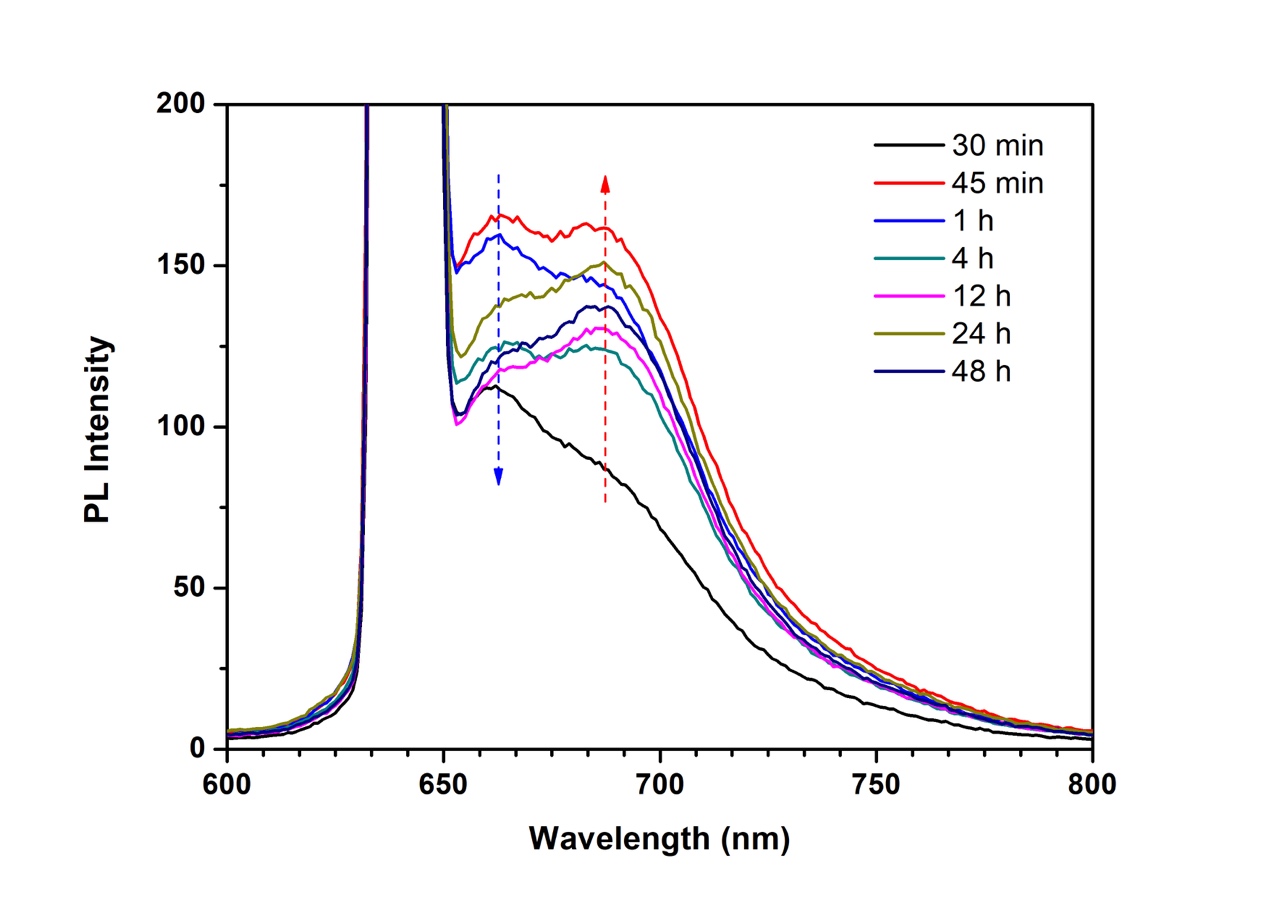


**Fig. S4.** Time-dependent fluorescence spectra of the reaction solution from Dex-*g*-OA FRET micelles in CH_2_Cl_2_/H_2_O.

**Table S2.** FRET ratio (*r*) at different time interval calculated from the fluorescence spectra in Fig. 3d and Fig. S4.

| Trial | Solvent | Time (h) | FRET ratio (*r*) |
| --- | --- | --- | --- |
| 1 | THF/H_2_O | 0.5 | 0.27 |
|  |  | 1.0 | 0.39 |
|  |  | 4.0 | 0.47 |
|  |  | 8.0 | 0.49 |
|  |  | 24.0 | 0.51 |
| 2 | CH_2_Cl_2_/H_2_O | 0.5 | 0.41 |
|  |  | 1.0 | 0.46 |
|  |  | 4 | 0.48 |
|  |  | 12 | 0.51 |
|  |  | 24 | 0.51 |
|  |  | 48 | 0.52 |


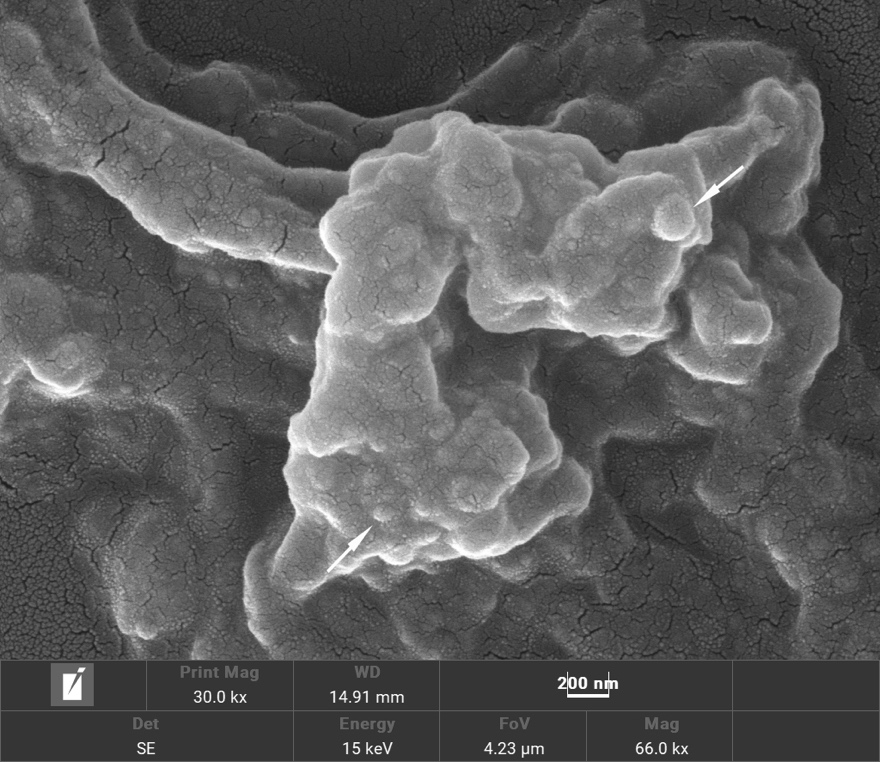


**Fig. S5.** SEM image of the Fe_3_O_4_@Dex-*g*-OA nanocomposites prepared with Dex-CHO/Fe_3_O_4_ mass ratio of 3/1.

**Table S3.** The Zeta potential (mV) of Fe_3_O_4_@Dex-*g*-OA nanocomposites at different time intervals.

| Time (h) | 2 | 12 | 24 | 26 | 108 | 180 |
| --- | --- | --- | --- | --- | --- | --- |
| M1 | -32.3±0.3 | -31.1±1.1 | -29.1±0.8 | -25.3±0.8 | -22.5±0.9 | -20.5±0.7 |
| M2 | -25.1±1.6 | -22.5±1.1 | -20.9±0.4 | -19.0±2.2 | -16.4±1.3 | -16.8±0.2 |
| M3 | -22.1±1.8 | -21.2±0.6 | -14.7±0.1 | -13.3±0.1 | -18.8±0.2 | -18.8±0.6 |
| M4 | -22.1±1.6 | -17.4±0.8 | -13.7±0.2 | -10.5±0.4 | -17.6±0.6 | -17.5±0.6 |
| M5 | -15.5±0.2 | -16.6±0.1 | -14.8±0.3 | -13.5±0.4 | -16.3±0.3 | -13.3±0.3 |


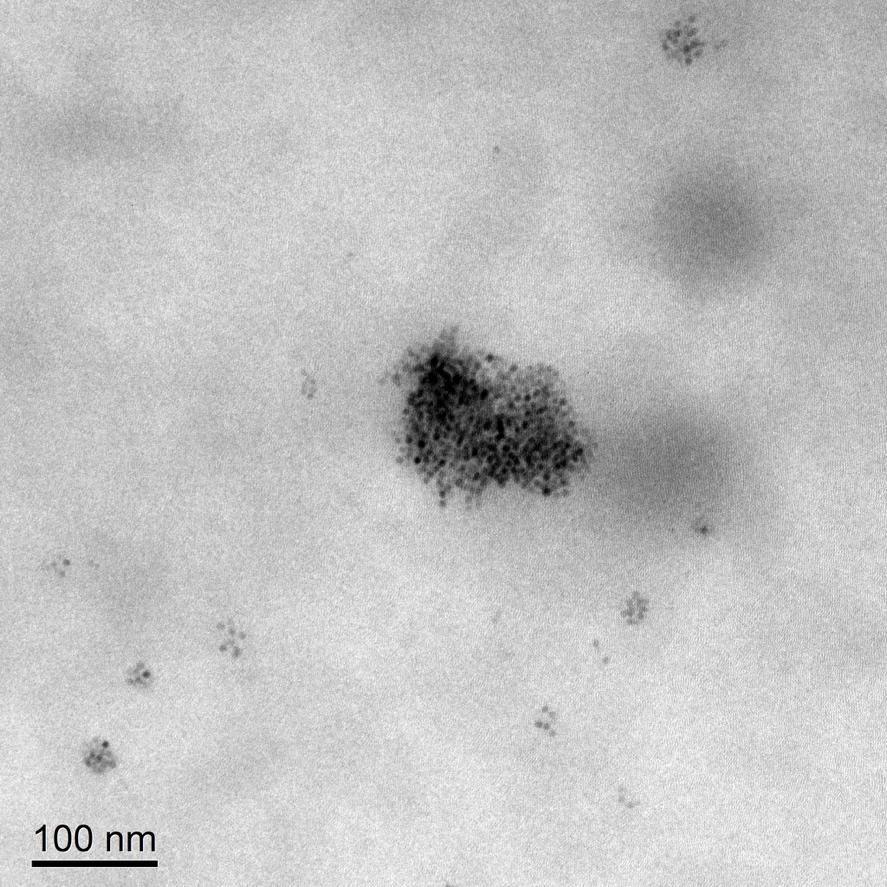


**Fig. S6.** TEM image of the Fe_3_O_4_@Dex-*g*-OA nanocomposites prepared with Dex-CHO/Fe_3_O_4_ mass ratio of 100/1.


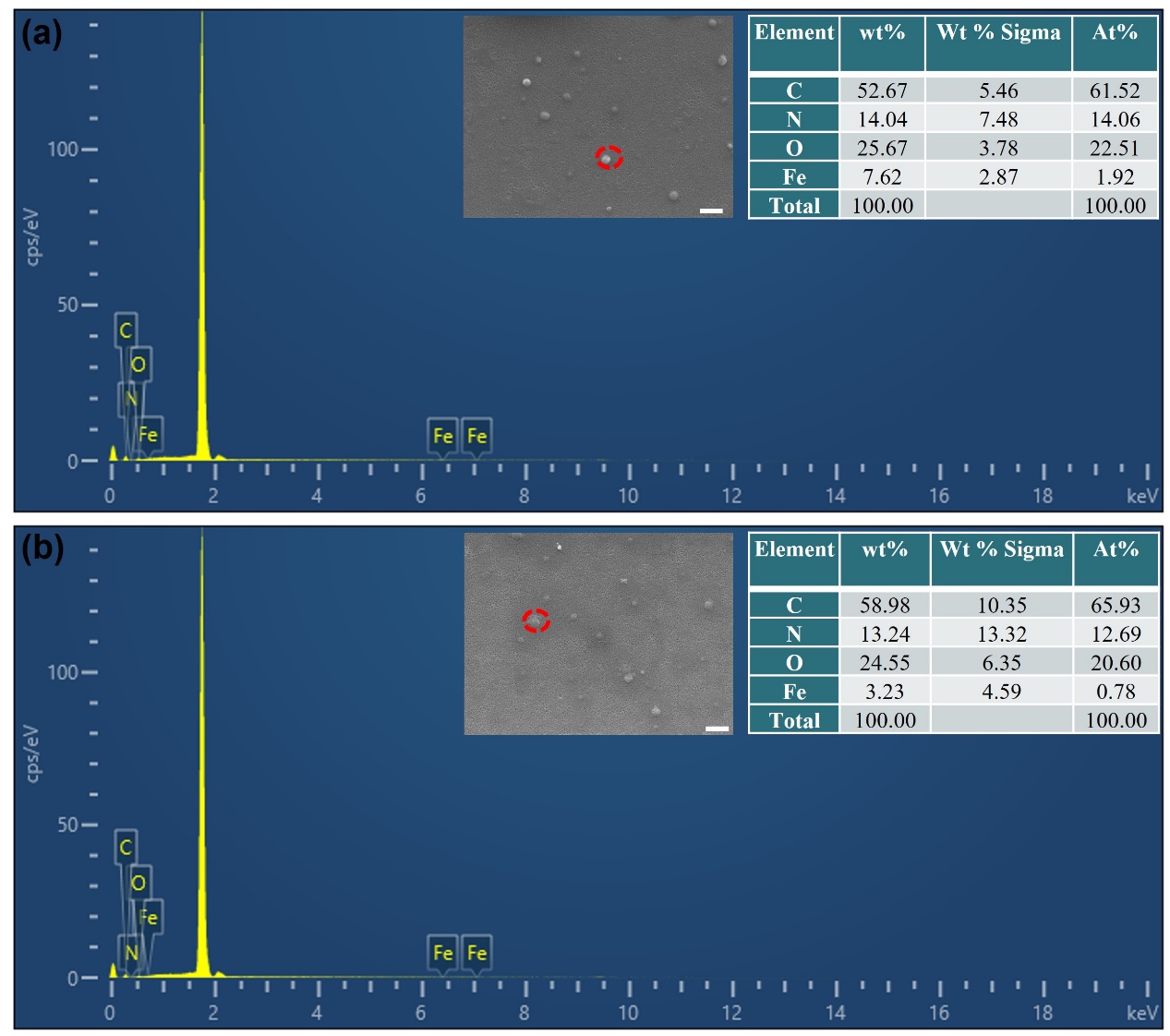


**Fig. S7.** SEM and EDS images of the Fe_3_O_4_@Dex-*g*-OA nanocomposites prepared with Dex-CHO/Fe_3_O_4_ mass ratio of 5/1 (a) and 7/1 (b). (Scale bar: 500nm)

**
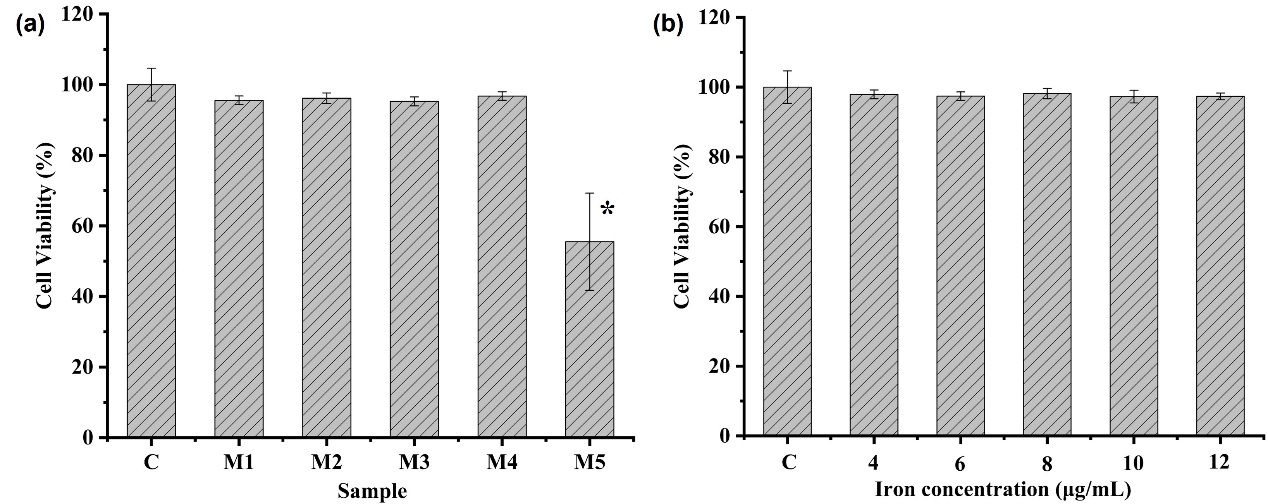
**

**Fig. S8.** Cell viability profile of Fe_3_O_4_@Dex-*g*-OA nanocomposites at the Fe concentration of 10 μg/mL (a) and M3 treated HepG2 cells at different Fe concentration (b). (n=4; C: control; *: significantly different from the control group though statistical analysis, p < 0.01)
